# Multiple Functions of ATG8 Family Proteins in Plant Autophagy

**DOI:** 10.3389/fcell.2020.00466

**Published:** 2020-06-10

**Authors:** Fan Bu, Mingkang Yang, Xu Guo, Wei Huang, Liang Chen

**Affiliations:** ^1^State Key Laboratory for Conservation and Utilization of Subtropical Agro-Bioresources, College of Life Sciences, South China Agricultural University, Guangzhou, China; ^2^Guangdong Provincial Key Laboratory of Protein Function and Regulation in Agricultural Organisms, College of Life Sciences, South China Agricultural University, Guangzhou, China

**Keywords:** autophagy, ATG8, cargo receptors, selective autophagy, AIM, UIM

## Abstract

Autophagy is a major degradation process of cytoplasmic components in eukaryotes, and executes both bulk and selective degradation of targeted cargos. A set of autophagy-related (ATG) proteins participate in various stages of the autophagic process. Among ATGs, ubiquitin-like protein ATG8 plays a central role in autophagy. The ATG8 protein is conjugated to the membrane lipid phosphatidylethanolamine in a ubiquitin-like conjugation reaction that is essential for autophagosome formation. In addition, ATG8 interacts with various adaptor/receptor proteins to recruit specific cargos for degradation by selective autophagy. The ATG8-interacting proteins usually contain the ATG8-interacting motif (AIM) or the ubiquitin-interacting motif (UIM) for ATG8 binding. Unlike a single ATG8 gene in yeast, multiple ATG8 orthologs have been identified in the plant kingdom. The large diversity within the ATG8 family may explain the various functions of selective autophagy in plants. Here, we discuss and summarize the current view of the structure and function of ATG8 proteins in plants.

## Introduction

**FIGURE 1:**
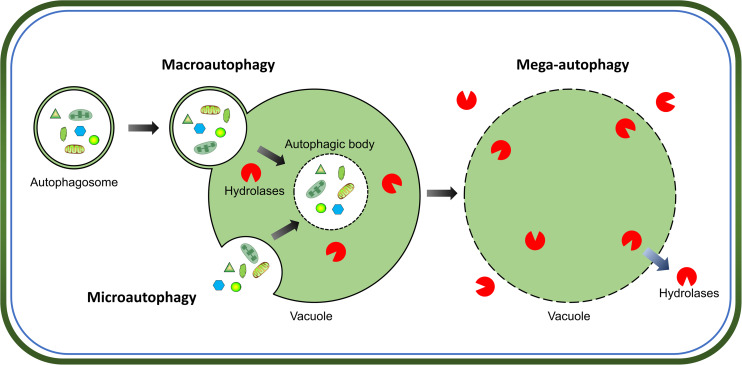
Morphological characteristics of three types of autophagy in plants. Microautophagy is achieved via invagination of the tonoplast to engulf cytoplasmic components into the vacuole. Macroautophagy delivers target cytoplasmic constituents through autophagosomes which fuse with the tonoplast to release internal vesicle into the vacuole. Both microautophagy and macroautophagy involve the formation of autophagic body and degradation of cargos by hydrolases in the vacuole. Additionally, mega-autophagy is the most extreme form of autophagy which involves permeabilization or rupture of the tonoplast and release of vacuolar hydrolases into the cytoplasm to degrade cytoplasmic materials *in situ*.

Intracellular protein quality-control is crucial for successful cell growth and development, which requires a proper balance between protein synthesis and degradation. The two major pathways for protein quality control in eukaryotes are, autophagy and the ubiquitin-proteasome system. Autophagy is the main process for the degradation of long-lived cytosolic proteins and organelles; whereas the ubiquitin-proteasome system is responsible for the degradation of short-lived proteins (Klionsky and Emr, 2000). Three types of autophagy have been reported in plants: microautophagy, macroautophagy, and mega-autophagy (Marshall and Vierstra, 2018). During microautophagy, cytoplasmic components are engulfed by invagination of the tonoplast into the vacuole; in contrast, macroautophagy involves the trapping of target cytoplasmic constituents double-membrane vesicles called autophagosomes, in which they are delivered to the vacuole for degradation (Figure 1). Macroautophagy is the predominant, and most studied form of autophagy in plants. This process involves the sequestration of cytoplasmic materials by a double-membrane vesicle called autophagosome, which delivers the intracellular cargo into the vacuole for degradation (Figure 1). Mega-autophagy is the most extreme form of autophagy which occurs at the final stage of programmed cell death (PCD). During mega-autophagy, the tonoplast permeabilizes and ruptures, and vacuolar hydrolases are released into the cytoplasm to degrade cytoplasmic components (Figure 1).

Macroautophagy (hereafter referred to as autophagy) plays an important role in plant growth and development, as well as in various biotic and abiotic stress responses (Liu and Bassham, 2012; Signorelli et al., 2019). Under normal conditions, autophagy remains at a basal level for maintaining cellular homeostasis. Although autophagy-deficient mutants are not lethal, lack of autophagy leads to premature leaf senescence as well as reduced seed yield and quality in plants (Phillips et al., 2008; Chung et al., 2010; Barros et al., 2017). In contrast, enhanced autophagy, which is caused by the overexpression of *ATG* genes, improves plant growth, seed yield, and nitrogen remobilization efficiency (Minina et al., 2018; Chen et al., 2019). To cope with various stresses, autophagy is upregulated to promote plant survival (Signorelli et al., 2019; Su et al., 2020). During senescence or stress responses, autophagy contributes to the recycling of cellular material and remobilization of nutrients, including proteins, lipids, carbohydrates (Masclaux-Daubresse et al., 2017).

**FIGURE 2:**
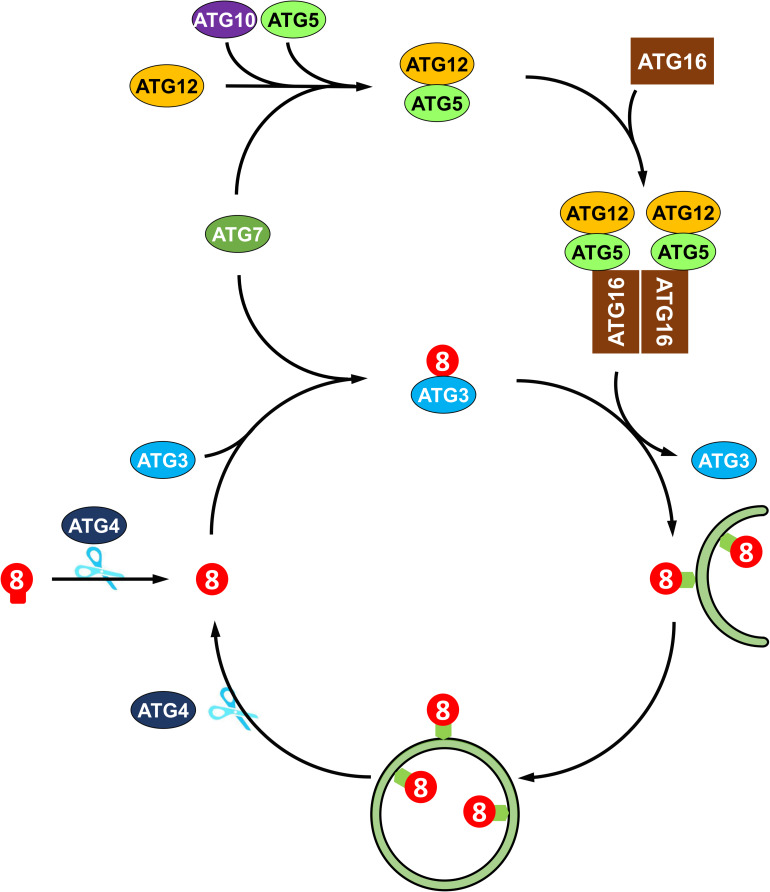
Recycling of ATG8 protein in plant autophagy. ATG8 protein is synthesized as proprotein, which is cleaved by the cysteine protease Atg4 to expose a glycine residue at the C-terminus. Then, the processed ATG8 protein is conjugated to phosphatidylethanolamine (PE) by E2-like ATG3 and the E3-like ATG12-ATG5-ATG16 complex. The ATG8-PE adduct coats the expanding phagophore and contributes to the maturation of autophagosome. Again, ATG8-PE attached to the outer autophagosome membrane is deconjugated from PE by ATG4, thus recycling ATG8 protein.

The molecular mechanisms of autophagy are sophisticated and involve a number of autophagy-related proteins that participate in autophagosome formation. ATGs can be divided into several functional groups: the ATG1 kinase complex, the ATG9 recycling complex, the phosphatidylinositol 3-kinase (PI3K) complex, and two ubiquitin-like conjugation systems, namely, the ATG8 lipidation system and the ATG12 conjugation system (Li and Vierstra, 2012). In Arabidopsis, the ATG1 kinase complex, consisting of ATG1, ATG13 and two additional subunits, ATG11 and ATG101, regulates the induction of autophagy (Suttangkakul et al., 2011; Li et al., 2014). The transmembrane protein ATG9, along with ATG2 and ATG18, recruits lipids to the expanding phagophore (Zhuang et al., 2017, 2018). The PI3K complex, including VPS34, VPS15, VPS38, and ATG6, decorates the phagophore with phosphatidylinositol-3-phosphate (PI3P), which is essential for the vesicle nucleation in *Arabidopsis* (Zhuang et al., 2018). Two ubiquitin-like conjugation systems mediate the expansion of phagophore and autophagosome maturation (Marshall and Vierstra, 2018). In particular, ATG8 plays a central role in plant autophagy. As shown in Figure 2, newly synthesized ATG8 is cleaved by cysteine proteinase ATG4 to expose the C-terminal glycine residue (Yoshimoto et al., 2004). Subsequently, the exposed glycine of ATG8 is conjugated to the membrane lipid phosphatidylethanolamine (PE) in a ubiquitin-like conjugation reaction catalyzed by ATG7 (E1-like enzyme), ATG3 (E2-like enzyme) and the ATG12-ATG5 complex (E3-like enzyme) (Doelling et al., 2002; Thompson et al., 2005; Phillips et al., 2008; Chung et al., 2010). Moreover, the association of ATG8 with the autophagosome is a reversible process; thus, the ATG8-PE adduct can be deconjugated from the membrane by ATG4 proteinase, whereby released ATG8 is recycled to participate in a new conjugation reaction (Yoshimoto et al., 2004; Woo et al., 2014).

Autophagy was originally considered as a non-selective process for the bulk degradation of cytoplasmic components (Thompson and Vierstra, 2005). However, increasing evidence suggests that autophagy may also be a selective process involving the degradation of specific target cargos, such as proteins, protein aggregates, malfunctioning organelles, and even invading pathogens (Li and Vierstra, 2012; Marshall and Vierstra, 2018). ATG8 is crucial for both bulk and selective autophagy. During bulk autophagy, ATG8 is involved in phagophore membrane elongation and autophagosome assembly (Nakatogawa et al., 2007). As for selective autophagy, ATG8 plays an additional role in cargo selection by providing a docking site for numerous autophagy adaptors and receptors that recruit the target cargo for degradation (Johansen and Lamark, 2011). In this review, we analyze the structural features and interactions of ATG8 proteins in plants and address their fundamental roles in plant selective autophagy.

## Features of ATG8 Family Proteins in Plants

**FIGURE 3:**
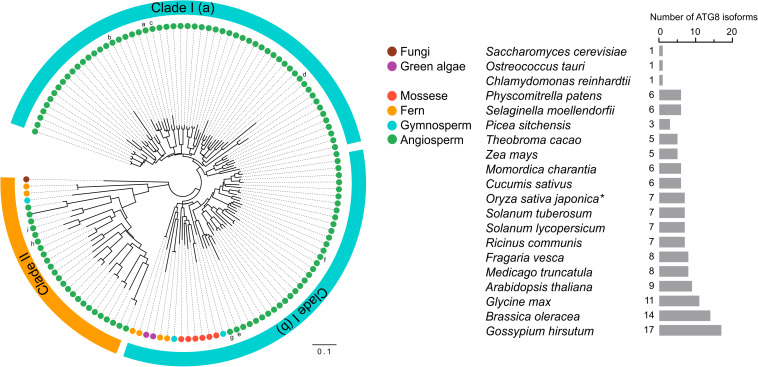
Phylogenetic analysis of ATG8s in plants. Phylogenetic analysis of ATG8 sequences from 19 plant species and *Saccharomyces cerevisiae* (budding yeast) with circular bands highlighting clades I and II. The tree was calculated in MEGA7 from a 419 nt alignment (ClustaW) of 133 sequences applying the maximum composite likelihood matrix. Colored dots indicate plant taxonomic groups. Nine *Arabidopsis thaliana* ATG8 variants AtATG8a-AtATG8i are shown in the tree. Branch lengths reflect the average number of substitutions per site. Scale bar: 0.01 substitutions per site. The number of ATG8 isoforms across the plant lineage is indicated in the right. The asterisk indicates OsATG8f and OsATG8i are excluded from phylogenetic analysis.

ATG genes are evolutionarily conserved across eukaryotes. Core ATG genes were originally identified in yeast, and most ATG genes have been found in plants based on sequence similarity with yeast homologs. Although yeast carries only one ATG8 gene, the plant ATG8 protein family diversifies from a single copy in algae to multiple genes in higher plants (Supplementary Table 1 and Figure 3). As shown in Supplementary Table 1, nine ATG8 isoforms have been identified in Arabidopsis, five in maize, seven in rice and potato, and eleven in soybean (Hanaoka et al., 2002; Chung et al., 2009; Perez-Perez and Crespo, 2010; Xia et al., 2011, 2012; Maqbool et al., 2016; Kellner et al., 2017; Zess et al., 2019). However, early-diverged plant lineages, such as algae, possess only a single ATG8 (Supplementary Table 1 and Figure 3). On the other hand, a recent study aiming to estimate ATG8 diversity across green plants found that, in order to adapt to adverse and complex conditions, the ATG8 gene family has undergone large expansion in plants via multiple whole-genome duplications (Kellner et al., 2017). As a result, ATG8 expansion may have led to the current diversity of selective autophagy in plants.

Plant ATG8 genes can be grouped into two clades by phylogenetic analysis (Figure 3). Clade I, which includes most of the plant ATG8 family members, is closely related to fungi, whereas clade II is more similar to the ATG8 homologs in animals (Kellner et al., 2017). However, some plant ATG8 isoforms in clade II, such as ATG8h and ATG8i in Arabidopsis, lack extra amino acid residues at the C-terminus after the glycine residue, which indicates that these ATG8 proteins can interact with the autophagosome membrane without ATG4 processing (Seo et al., 2016). In addition, clade I can be further divided into two subgroups, clade I (a) and clade I (b), containing AtATG8a-d and AtATG8e-g, respectively (Figure 3), which is consistent with the results of previous studies (Seo et al., 2016; Kellner et al., 2017). However, there is a slight difference in gene classification compared with that reported by Slavikova et al. (2005), in which AtATG8a, AtATG8c, AtATG8d, and AtATG8f were grouped together, while AtATG8b, AtATG8e, and AtATG8g were grouped together.

**FIGURE 4:**
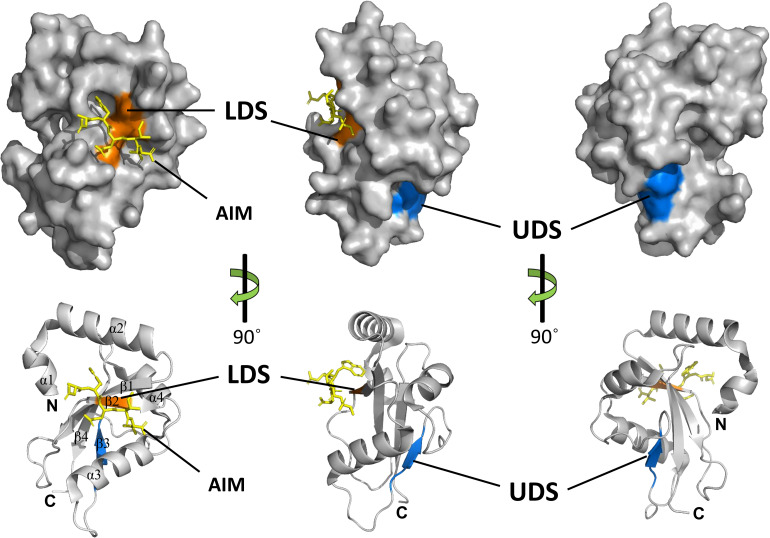
Structures and binding motifs of ATG8 proteins. The 3-dimensional structure of potato ATG8CL (PDB: 5L83) was downloaded from the Protein Data Bank (https://www.rcsb.org/pdb). The positions of LDS (orange) and UDS (blue) are mapped with the corresponding positions of yeast ATG8. The AIM-containing peptide (residues 377–381) from *Phytophthora infestans* PexRD54 is shown as a yellow stick. α-Helices, β-strands, N-terminal and the C-terminal regions are shown in ribbon diagrams.

Crystal structures of several ATG8 family proteins from yeast, animals and plants, have been solved (Sugawara et al., 2004; Kumeta et al., 2010; Maqbool et al., 2016). These studies indicated that ATG8 proteins conserved among eukaryotes contain an N-terminal helical domain and C-terminal ubiquitin domain (Figure 4). The C-terminal ubiquitin domain, similar with ubiquitin, adopts a β-grasp (ubiquitin-like) fold consisting of four β-strands (β1–β4) and two α helices (α3 and α4). However, the N-terminal helical domain is formed by two other α helices (α1 and α2), which is a unique feature of ATG8 proteins. The C-terminal ubiquitin domain is conserved among ATG8 family members and might play a crucial role in protein-protein interactions (Shpilka et al., 2011), whereas the N-terminal helical domain is not conserved and is responsible for binding specificity to ATG8-interacting proteins in animals and plants (Coyle et al., 2002; Ketelaar et al., 2004; Zess et al., 2019).

Generally, ATG8 binds to specific proteins with the ATG8-interacting motif (AIM) in yeast and plants, or the LC3-interacting region (LIR) in animals (Noda et al., 2010). The core AIM sequence is defined as W/F/Y-XX-L/I/V, an aromatic amino acid (Trp, Tyr, or Phe) followed by two random amino acids and an aliphatic amino acid (Leu, Ile, and Val). A hydrophobic patch of ATG8 known as the LIR/AIM docking site (LDS), with two distinct pockets (W and L pockets), is responsible for binding with AIM (Noda et al., 2010). The W pocket is formed at the interface between the β-grasp and the N-terminal helices and embraces the aromatic residue of AIM, while the L pocket is located in β-grasp fold and embraces the aliphatic amino acid of AIM (Maqbool et al., 2016).

Notably, there are some proteins that bind ATG8 differently from the AIM-LDS type of binding in animals and plants (Lin et al., 2013; Marshall et al., 2015). CoIP and pull-down assays demonstrated that SQST-1 (the p62 homolog in *Caenorhabditis elegans*) associated with LGG-1 (the ATG8 homolog in *C. elegans*), while mutating the LIR motif in SQST-1 had no effect on the interaction with LGG-1, which indicated that the LIR motif is not necessary for binding of SQST-1 to LGG-1 (Lin et al., 2013). Furthermore, ubiquitin receptor RPN10 (regulatory particle non-ATPase 10) acts as an autophagy receptor for degradation of the proteasome by binding to ATG8 in Arabidopsis. However, the interaction with ATG8 depends on the ubiquitin-interacting motif (UIM), not the canonical AIM (Marshall et al., 2015). Generally, UIM is a 20-amino acid stretch of the amino acid sequence that folds into a single α helix in which a consensus sequence (φ-θ-X-A-φ-X-X-S) for ATG8 binding is defined (where φ is a hydrophobic residue, θ is a hydrophilic residue, A is alanine, S is serine and X is any amino acid, respectively) (Marshall et al., 2019). ATG8 binds to proteins with a UIM through an alternative interaction site called UIM-docking site (UDS). The UDS sequence within ATG8 is highly conserved across species. Saturating mutagenesis identified a core 4-residue motif for permitted residues of φ-F-φ-Ω/T within UDS, where φ represents small hydrophobic residues and Ω represents aromatic residues (Marshall et al., 2019). In addition, the location of UDS is opposite to that of LDS; thus, ATG8 can bind to proteins with AIM and UIM simultaneously (Figure 4). The UIM-UDS interface greatly widens the range of autophagy receptors and adaptors, thereby expanding the scope of selective autophagy.

## Functions of the ATG8 Proteins in Plants

ATG8 proteins perform multiple functions in plants. For example, ATG8 proteins regulate membrane elongation during autophagosome biogenesis. Moreover, they play a crucial role in cargo recognition for selective autophagy by interacting with autophagy receptors/adaptors. In addition to their autophagic roles, ATG8 proteins play non-autophagic roles in plants. In this section, we summarize the current knowledge regarding the different roles of ATG8 proteins in plants.

## Autophagosome Biogenesis

ATG8 is a ubiquitin-like protein required for autophagosome formation. First, mature ATG8 processed by the ATG4 protease is conjugated to the membrane lipid PE in Arabidopsis (Xie et al., 2008; Woo et al., 2014). subsequently, the ATG8-PE adduct promotes autophagosome biogenesis through membrane tethering and remodeling (Ohsumi, 2001; Nakatogawa et al., 2007). On one hand, the loss of ATG8 functionality blocks autophagosome formation in yeast and other fungi (Kirisako et al., 1999; Ren et al., 2018); however, this phenotype has not been observed in plants yet, probably due to ATG8 gene redundancy. On the other hand, overexpression of ATG8 promotes autophagosome formation in Arabidopsis and rice (Chen et al., 2019; Yu et al., 2019). In addition to ATG8 lipidation, ATG8 proteins are deconjugated by ATG4 and released from the autophagosome membrane, whereby the released ATG8 proteins appear to be recycled to facilitate autophagosome formation in plants (Yoshimoto et al., 2004). In addition to supplying ATG8 free protein, the delipidation of ATG8-PE also plays an important role in autophagosome membrane expansion (Hirata et al., 2017). Indeed, the inability for ATG8 delipidation results in mislocalization of the autophagosome to the vacuolar membrane and in defective autophagosome biogenesis (Nair et al., 2012). Therefore, the ATG4-mediated deconjugation of ATG8-PE plays a dual role in autophagosome biogenesis, namely, during the early stage of autophagosome formation, the release of ATG8 from non-autophagosomal membranes supplies the increasing demand for ATG8, while at the later stage, the release of ATG8 from the phagophore membrane facilitates autophagosome maturation (Yu et al., 2012). In addition to inducing autophagy at the early stage in autophagosome biogenesis, ATG8 downregulates autophagy at the later stage. The ATG1/13 kinase complex plays an essential role in the initiation of autophagy across the evolutionary scale from yeast to plants (Mizushima, 2010; Suttangkakul et al., 2011). ATG8 directly binds ATG1 and ATG11 in an AIM-dependent manner. Moreover, ATG8-binding triggers self-digestion of the ATG1-ATG13 complex, thereby suppressing autophagic activity (Suttangkakul et al., 2011; Li et al., 2014).

In addition to its interaction with ATG proteins, ATG8 promotes phagophore expansion and maturation by recruiting a non-ATG protein, SH3 DOMAIN-CONTAINING PROTEIN2 (SH3P2) in Arabidopsis. During autophagy induction, SH3P2 is recruited to the phagophore assembly site (PAS) by membrane associated ATG8 protein; then, ATG8 binds to phosphatidylinositol 3-phosphate (PI3P) and coordinates with the PI3K complex to facilitate autophagosome formation. Consistently, knockdown of SH3P2 significantly suppresses autophagosome formation (Zhuang et al., 2013).

## Autophagic Cargo Recognition

**FIGURE 5:**
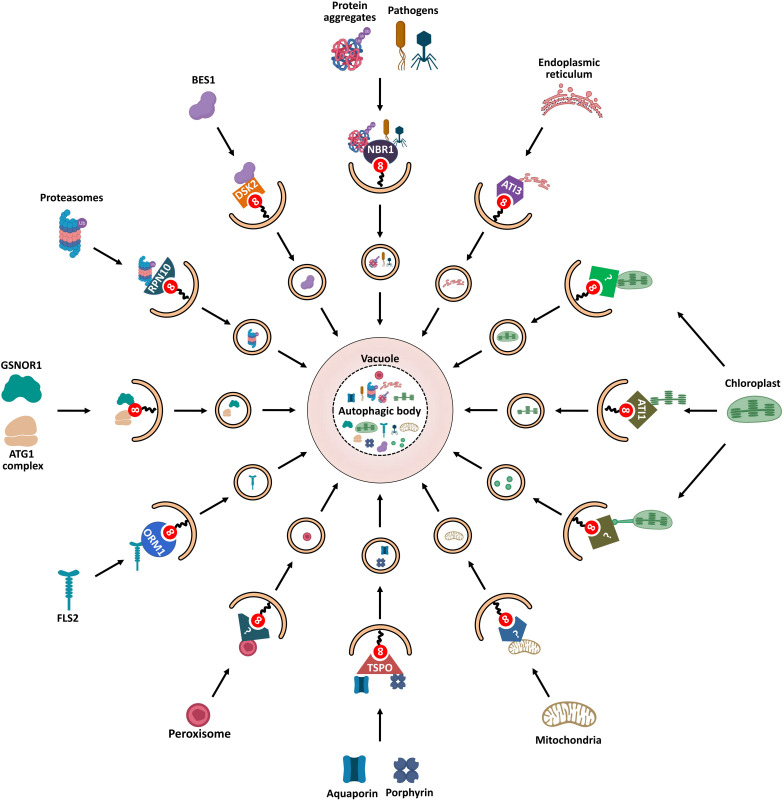
Schematic diagram of ATG8-mediated selective autophagy pathways in plants. Various ATG8-binding proteins act as selective autophagy receptors and mediate the delivery of specific target cargos to autophagosomes for degradation. NBR1, the first autophagy receptor identified in plants, mediates selective degradation of ubiquitinated protein aggregates and virulence factors; degradation of chloroplasts is mediated by three autophagic pathways: First, ATI1 binds and transports thylakoid proteins to vacuole for autophagic degradation through ATI1-PS bodies; Then, stromal components including Rubisco are engulfed by autophagic membrane to form Rubisco-containing bodies (RCBs) that are delivered to vacuole for degradation; Finally, the whole chloroplasts are entirely engulfed by the vacuolar membrane for degradation. ATG11 is involved in the autophagic degradation of mitochondria; however, the cargo receptor of mitophagy remains unknown. PEX6 and PEX10 may function as potential pexophagy receptors. In turn, degradation of the endoplasmic reticulum is mediated by another ATI protein, ATI3. RPN10 is involved in selective degradation of inactivated proteasomes extensively ubiquitylated. The selective autophagic degradation of BR signaling pathway transcription-factor BES1 is mediated by DSK2. Aquaporin PIP2;7 and porphyrin (heme) are both recognized by receptor TSPO and subjected to selective autophagic degradation. ORM1/2 proteins mediate the degradation of plant immune receptor FLS2. In addition to cooperating with autophagy receptors, some AIM/UIM-containing proteins, such as ATG1, ATG11, and GSNOR1, also bind directly to ATG8 to mediate their degradation.

Autophagy was initially thought to be an non-selective, bulk degradation process of cytoplasmic contents. However, increasing evidence has shown that autophagic cargos can be selectively targeted for degradation. Subsequently, selective autophagy has been characterized according to the type of targeted substrates, such as proteins (proteaphagy), protein aggregates (aggrephagy), pathogens (xenophagy), chloroplasts (chlorophagy), endoplasmic reticulum (reticulophagy), and others. In plants, ATG8 proteins play a crucial role in selective autophagy through their interaction with various AIM/UIM-containing proteins (Marshall and Vierstra, 2018; Lei and Klionsky, 2019). Recently, an increasing number of ATG8-binding proteins, many of which are likely cargo receptors, have been identified in plants. As shown in Figure 5, these proteins bind both autophagic cargos and ATG8 proteins to facilitate cargo gathering. Therefore, the association of ATG8 proteins with adaptor/receptor proteins is necessary for cargo selection and degradation. Here, ATG8-interacting receptors and cargos that are essential for selective autophagy in plants are discussed.

### NBR1

Protein aggregates are degraded through the ubiquitin-proteasome system (UPS) and autophagy (termed aggrephagy) to maintain cellular homeostasis (Lamark and Johansen, 2012). In animals, two selective cargo receptors, p62 and NBR1 (neighbor of BRCA1 gene 1), are responsible for autophagic degradation of ubiquitinated protein aggregates (Lamark et al., 2009); p62 was first identified as an autophagic receptor to bind both target-associated ubiquitin and LC3 (Pankiv et al., 2007). Like p62, NBR1 was shown to be an Ub- and LC3-binding protein (Kirkin et al., 2009). Both p62 and NBR1 are structurally similar, containing an N-terminal PB1 domain, a LIR motif that can interact with LC3, and a C-terminal ubiquitin-associated (UBA) domain that can bind ubiquitin (Lamark et al., 2009).

AtNBR1, the first selective autophagy receptor reported in plants, is a hybrid protein homolog to both NBR1 and p62 that harbors functions of these two proteins (Svenning et al., 2011). AtNBR1 binds ubiquitin through a C-terminal UBA domain and interacts with various ATG8 proteins via an evolutionarily conserved AIM, mediating the sequestration of ubiquitinated protein aggregates into autophagosomes (Svenning et al., 2011; Jung et al., 2019). Joka2, a homolog of AtNBR1, has been identified as a selective autophagy receptor in tobacco (*Nicotiana tabacum*) (Zientara-Rytter et al., 2011). Similar to *atg* mutants, ubiquitinated protein aggregates are highly accumulated in both Arabidopsis and tomato *nbr1* mutant under heat stress and non-stress conditions (Zhou et al., 2013, 2014; Jung et al., 2019). These results suggest that NBR1 is an autophagy receptor involved in the selective degradation of protein aggregates in plants.

In addition to its role in aggrephagy, NBR1 acts as a xenophagy receptor in plant immune responses. For example, during viral infections, AtNBR1 contributes to plant immunity by directly binding the viral capsid protein P4 and particles of the cauliflower mosaic virus (CaMV), and the viral silencing suppressor HCpro of the turnip mosaic virus (TuMV); thus, mediating their selective autophagic degradation (Hafrén et al., 2017, 2018). Further, upon bacterial infection, NBR1-dependent selective autophagy enhances host antibacterial immunity by promoting autophagic degradation of an unknown factor required for bacterial pathogenesis and infection (Üstün et al., 2018). Surprisingly, plant pathogens can manipulate the host autophagy machinery to promote infection. Thus, Phytophthora infestans effector protein PexRD54 binds potato ATG8CL to prevent interaction of ATG8CL with the autophagy cargo receptor Joka2, thereby facilitating autophagic clearance of plant or pathogen proteins that negatively affect immunity (Dagdas et al., 2016; Maqbool et al., 2016). Meanwhile, PexRD54 activates autophagy to redistribute nutrients in favor of the pathogen (Dagdas et al., 2016).

### TSPO

Another autophagy receptor, TSPO (tryptophan-rich sensory protein), is a porphyrin-binding membrane protein which contains an AIM for ATG8 binding in Arabidopsis (Vanhee et al., 2011). AtTSPO is induced by abscisic acid (ABA) and abiotic stress such as osmotic or salt stress (Guillaumot et al., 2009; Balsemão-Pires et al., 2011). Thus, for example, the level of heme increases as a result of stress response and extra unbound heme may induce ROS production. In such case, TSPO shows a high affinity for heme and acts as a heme scavenger via ATG8-mediated selective autophagy, thus regulating heme levels in Arabidopsis cells (Vanhee et al., 2011). This result suggests that TSPO might modulate redox homeostasis through heme binding and scavenging during stress. Moreover, TSPO binds to the plasma membrane aquaporin PIP2;7 (plasma membrane intrinsic protein 2;7) for autophagic degradation (Hachez et al., 2014). This TSPO-mediated selective degradation of PIP2;7 has been proposed as a mechanism to protect plant cells from water deficit.

### RPN10

The UPS and autophagy are the two major protein quality-control pathways responsible for cellular homeostasis (Dikic, 2017). Ubiquitination serves as the degradation signal in both UPS and autophagy. Thus, UPS and autophagy functionally interconnect with each other (Ji and Kwon, 2017). Moreover, the 26S proteasome can be selectively degraded by autophagy (termed proteaphagy), which was first discovered in Arabidopsis (Marshall et al., 2015). Proteaphagy is induced by nitrogen starvation and proteasome inhibition. When 26S proteasomes are inactivated by a proteasome inhibitor or by a genetic mutation, proteasomes are extensively ubiquitylated and selectively degraded by autophagy mediated by the proteasome subunit RPN10. RPN10 does not contain the canonical AIM as other autophagy receptors but harbors three UIMs, in which UIM1 is responsible for binding ubiquitin and UIM2 for binding ATG8. Upon inhibitor-induced proteaphagy, ubiquitylated proteasomes are recruited by the free form of RPN10 through UIM1; meanwhile, RPN10 also binds membrane-associated ATG8 by UIM2 to form a stable tripartite complex which is engulfed in autophagosomes and then degraded (Marshall et al., 2015). The interaction between RPN10 and ATG8 is necessary for inhibitor-induced proteaphagy and is highly conserved across plant species. However, this binding is absent in yeast and animals because the orthologs of RPN10 in yeast and humans lack the UIM-related ATG8-binding motif. Alternatively, yeast proteaphagy employs a CUE-domain protein, namely, Cue5, as a ubiquitin-ATG8 adaptor (Marshall et al., 2016); Cue5 simultaneously binds ATG8 and ubiquitin through the AIM and the CUE domain, respectively (Lu et al., 2014).

### ATI Proteins

Five plant-specific ATG8-interacting (ATI) proteins were found to interact with ATG8 in Arabidopsis. These proteins can be divided into two groups, ATI1/2 and ATI3a/b/c (Honig et al., 2012; Zhou et al., 2018). ATI1 and ATI2 are transmembrane proteins which contain two putative AIMs. However, only the N-terminal AIMs are responsible for interacting with ATG8 (Sjogaard et al., 2019). Meanwhile, ATI1 also interacts with chloroplast-associated proteins, such as NPQ4 and APE1 (Honig et al., 2012; Michaeli et al., 2014). Upon carbon starvation, the ATI1-decorated plastid-associated bodies (ATI1-PS bodies) deliver chloroplast components including stromal proteins, envelope, and thylakoid proteins to the vacuole for degradation, which is referred as chlorophagy (Michaeli et al., 2014). The interaction between ATI1 and ATG8 contributes to the targeting of the ATI1-PS bodies to the autophagosomes (Michaeli et al., 2014). Interestingly, ATI1 bodies are mainly localized in endoplasmic reticulum (ER)-associated vesicles and are distinct from autophagosomes (Honig et al., 2012). To date, whether ATI1 is involved in the selective autophagic degradation of ER (reticulophagy) remains unknown.

Additionally, ATI3 was identified as a specific receptor of reticulophagy in dicot plant species (Zhou et al., 2018). Three related ATI3 proteins (ATI3a/b/c) were found in Arabidopsis, all of which contain a C-terminal LIR motif and interact with ATG8. Additionally, ATI3a interacts with ER-localized UBAC2a/b (Ubiquitin associated proteins 2a/b), which are involved in ER-associated degradation (Zhou et al., 2018). Further research has shown that NAI2, an ER body component, interacts with an unknown protein encoded by the *At4g15545* gene, which is a potential UBAC2-interacting protein (Wang et al., 2019). These results suggest that ATI3 plays an important role in selective autophagy degradation of ER components.

### ORM Proteins

Orosomucoid (ORM) proteins are known as negative regulators of sphingolipid biosynthesis (Breslow et al., 2010). A recent study reported that ORM proteins act as selective autophagy receptors to mediate the degradation of plant immune receptor FLS2 (FLAGELLIN-SENSING 2) (Yang et al., 2019). Two ORM proteins, ORM1 and ORM2, were identified in Arabidopsis (Li et al., 2016). ORM1 contains an N-terminal AIM, and ORM2 contains two AIMs at both N and C terminuses. ORM proteins bind both ATG8 and FLS2 simultaneously. Furthermore, ORM downregulation increases FLS2 accumulation and FLS2-dependent immune responses, while overexpression of ORM causes FLS2 degradation and suppression of FLS2-dependent signaling (Yang et al., 2019). These results suggest that ORM-mediated selective autophagy plays a key role in plant immunity.

### DSK2

DSK2 (dominant suppressor of KAR2) is a ubiquitin-binding receptor protein related to protein degradation pathways in eukaryotes (Lee and Brown, 2012). Two DSK2 proteins (DSK2A and DSK2B) were identified in Arabidopsis (Farmer et al., 2010). AtDSK2 acts as an autophagy receptor for transcription factor BES1 (BRI1-EMS suppressor 1) of the brassinosteroid (BR) pathway. Under drought and starvation conditions, BES1 is ubiquitinated by the E3 ubiquitin ligase SINAT2 (SEVEN-*IN ABSENTIA* 2), which promotes binding of BES1 to DSK2. Concomitantly, DSK2 is phosphorylated by kinase BIN2, which enhances the interaction between DSK2 and ATG8. Thus, during abiotic stress, DSK2 recruits BES1 to the ATG8-located autophagosomes for degradation. DSK2 decreases BR signals by selective degradation of BES1 through autophagy to switch plant metabolism from growth to stress mode (Nolan et al., 2017).

### Uncertain Receptor in Several Types of Selective Autophagy

Chlorophagy is important for quality control and nutrients recycling (Jarvis and López-Juez, 2013). Besides ATI1-PS body, four other types of chlorophagy are reported in plants (Zhuang and Jiang, 2019). However, the receptors for these types of chlorophagy are undetermined. First, the whole chloroplasts are captured by autophagic vesicles and delivered to the vacuole for degradation upon ultraviolet radiation (Izumi et al., 2017). In addition, the second pathway for whole chloroplasts degradation is mediated by microautophagy (Nakamura et al., 2018). High-intensity light triggers chloroplast envelope damage and leads to chloroplast swelling, These swollen chloroplasts are directly engulfed by the vacuolar membrane and degraded by vacuolar hydrolase, which is dependent on the core autophagic machinery. The third type of chlorophagy is mediated by rubisco-containing bodies (RCB). During leaf senescence, RCBs containing chloroplast stromal proteins including Rubisco are transported to the vacuolar lumen for recycling through an autophagy-dependent manner (Ishida et al., 2008; Izumi et al., 2010).

The autophagic degradation of mitochondria (mitophagy) is well described in mammals. However, the understanding of mitophagy is much less in plants. A recent study reported that Arabidopsis ATG11 plays an important role in plant mitophagy (Li et al., 2014). Arabidopsis ATG11 interacts with ATG13, ATG101, ATG8, and is colocalized with mitochondria (Li et al., 2014). The turnover of mitochondrial proteins is blocked in Arabidopsis *atg11* mutants (Li et al., 2014). However, the mechanism of ATG11-mediated plant mitophagy is still unknown, and the cargo receptor of plant mitophagy is still waiting to be discovered.

Peroxisomes participate in various cellular processes in plants, such as lipid metabolism, photorespiration, and phytohormone synthesis. Pexophagy, the autophagic degradation of peroxisomes, is important for peroxisome quality control. The receptor of plant pexophagy remains undetermined. Atg30 and Atg36, two AIM-containing proteins, have been identified as pexophagy receptors in yeast (Farre et al., 2013; Zientara-Rytter et al., 2018). However, homologs of Atg30 and Atg36 are not found in plants. NBR1, together with p62, acts as a pexophagy receptor to recruit peroxisomes to PAS in mammals (Deosaran et al., 2013; Zhang et al., 2015; Sargent et al., 2016). Although plant NBR1 has been demonstrated as a receptor in aggrephagy and xenophagy, there is no direct evidence indicating that NBR1 is involved in pexophagy in plants. However, a recent study reported that Arabidopsis PEX6 (Peroxin 6) and PEX10 interact with ATG8, suggesting that they may function as potential pexophagy receptors in plants (Xie et al., 2016).

### Receptor-Independent Cargo Recognition

In addition to cooperating with autophagy receptors, ATG8 proteins directly bind to the substrates to mediate their degradation. For instance, *Nicotiana benthamiana* ATG8f protein was demonstrated to target virulence protein βC1 of CLCuMuV (cotton leaf curl Multan virus) for autophagic degradation without the help of a cargo receptor such as NBR1/Joka2 (Haxim et al., 2017). This finding indicates that autophagy contributes to plant immunity through selective degradation of viral proteins independently of canonical autophagy receptors. Another example is NO-induced selective autophagy of GSNOR1 (*S*-nitrosoglutathione reductase 1) during hypoxia (Zhan et al., 2018). GSNOR1, a master regulator of NO signaling, is stable under normal conditions. However, hypoxia induces conformational changes in GSNOR1, whereby it exposes the AIM motif and facilitates the interaction of GSNOR1 with ATG8, ultimately leading to the selective degradation of GSNOR1 (Zhan et al., 2018).

## Non-Autophagic Roles of ATG8 Proteins in Plants

In addition to their autophagic roles, ATG8 proteins fulfill functions that are not associated with autophagy. Thus, according to a recent study, MATE transporter-family protein ABS3 (ABNORMAL SHOOT 3) promoted senescence under both, normal and nutrient-deprived conditions in Arabidopsis (Jia et al., 2019). ABS3 contains two AIMs which are critical for binding to ATG8. Interestingly, this ATG8-ABS3 interaction is independent of the autophagic function of ATG8, but essential for ABS3-mediated senescence (Jia et al., 2019). In brief, ATG8 plays dual roles in controlling plant senescence. Under normal nutrient conditions, ATG8 is lipidated and activates autophagy to promote plant longevity. However, under nutrient-deprived or autophagy-deficiency conditions, ATG8 binds to ABS3 to promote ABS3 degradation and plant senescence independently of autophagy (Jia et al., 2019).

ATG8 proteins participate in diverse intracellular transport processes in animals (Shpilka et al., 2011). Animal ATG8 proteins can be divided into three subfamilies: microtubule-associated protein 1 light chain 3 (LC3), γ-aminobutyric acid receptor-associated protein (GABARAP) and Golgi-associated ATPase enhancer of 16 kDa (GATE-16). GABARAP is involved in the intracellular trafficking of membrane proteins, such as GABA, κ-opioid, transferrin receptors and *N*-cadherin/β-catenin complex (Green et al., 2002; Leil et al., 2004; Chen et al., 2007, 2011; Nakamura et al., 2008). GATE-16 participates in intra-Golgi transport of Golgi SNARE protein 28 (GOS-28) and *N*-ethylmaleimide sensitive factor (NSF) (Sagiv et al., 2000; Muller et al., 2002). LC3 binds to FYVE and coiled-coil domain containing protein 1 (FYCO1) that interacts with Rab7 to mediate autophagosome transport to the vacuole along microtubule (Pankiv et al., 2010). In plants, the role of ATG8 in intracellular transport processes is poorly understood. Although FYCO1 homologs have been identified in Arabidopsis (Wywial and Singh, 2010), it is still unknown whether FYCO1 proteins are involved in autophagosome transport in plants. The exocyst is an evolutionary conserved protein complex mediating early tethering of secretory vesicles to the plasma membrane during exocytosis. Most of Exo70 subunits in Arabidopsis possesses widespread AIMs (Tzfadia and Galili, 2013). Despite the lack of direct evidence for ATG8 binding, Exo70B1 is colocalized with the ATG8f in Arabidopsis (Kulich et al., 2013). Further, Exo70B2 has been demonstrated to interact with ATG8, an interaction enhanced by phosphorylation of MPK3 kinase that leads to autophagic recycling of Exo70B2 (Teh et al., 2018). These results suggest that Exo70B1 and Exo70B2 may be involved in autophagic transport into the vacuole.

## Conclusion and Prospects

Autophagy serves as an important catabolic mechanism involved in plant growth and development, and plant responses to stress. Initially, autophagy was known exclusively as a non-selective degradation process; however, increasing evidence suggests that autophagy is also a highly selective pathway to target specific substrates for degradation. ATG8 proteins play multifunctional roles in plant autophagy, promoting autophagosome biogenesis. Moreover, ATG8 proteins interact with various adaptor/receptor proteins to recruit specific targeted cargos for degradation through selective autophagy. The identification of ATG8-interacting autophagy receptor proteins helps us to understand how autophagy substrates are selected for degradation. Future identification of AIM/UIM-containing proteins should greatly expand the scope of selective autophagy. In addition, ATG8 is also involved in other intracellular processes that appear to be independent of autophagy. In conclusion, studies on ATG8 have greatly contributed to our understanding of the molecular basis for the connection of autophagy with other metabolic processes. Although extensive studies have been carried out on ATG8s in plants, there are many unanswered questions about their functions. An intriguing question relates to the presence of multiple ATG8 isoforms in plants, in contrast to a single ATG8 protein in yeasts; indeed, the reason for such great ATG8 diversity in plants remains unclear. Similarly, it is not known whether ATG8 isoforms interact with specific cargo receptors while engaging in different types of selective autophagy. In addition, the roles of ATG8 in intracellular trafficking are still poorly characterized in plants. Therefore, further study of the ATG8-interacting proteins will be important for understanding the role of ATG8s in autophagy-dependent and autophagy-independent functions.

## Author Contributions

LC conceptualized the review. FB, MY, and XG wrote the first draft. WH and LC critically revised the manuscript. All authors read and approved the final content.

## Conflict of Interest

The authors declare that the research was conducted in the absence of any commercial or financial relationships that could be construed as a potential conflict of interest.

## References

[B1] Balsemão-Pires E. Jaillais Y. Olson B. J. S. C. Andrade L. R. Umen J. G. Chory J. (2011). The Arabidopsis translocator protein (AtTSPO) is regulated at multiple levels in response to salt stress and perturbations in tetrapyrrole metabolism. *BMC Plant Biol.* 11:108. 10.1186/1471-2229-11-108 21689410 PMC3141639

[B2] Barros J. A. S. Cavalcanti J. H. F. Medeiros D. B. Nunes-Nesi A. Avin-Wittenberg T. Fernie A. R. (2017). Autophagy deficiency compromises alternative pathways of respiration following energy deprivation in *Arabidopsis thaliana*. *Plant Physiol.* 175 62–76. 10.1104/pp.16.01576 28710132 PMC5580740

[B3] Breslow D. K. Collins S. R. Bodenmiller B. Aebersold R. Simons K. Shevchenko A. (2010). Orm family proteins mediate sphingolipid homeostasis. *Nature* 463 1048–1053. 10.1038/nature08787 20182505 PMC2877384

[B4] Chen C. Wang Y. Huang P. Liu-Chen L.-Y. (2011). Effects of C-terminal modifications of GEC1 Protein and γ-Aminobutyric Acid Type A (GABAA) Receptor-associated Protein (GABARAP), two microtubule-associated proteins, on κ opioid receptor expression. *J. Biol. Chem.* 286 15106–15115. 10.1074/jbc.M111.230896 21388957 PMC3083202

[B5] Chen Q. Soulay F. Saudemont B. Elmayan T. Marmagne A. Masclaux-Daubresse C. L. (2019). Overexpression of ATG8 in *Arabidopsis* stimulates autophagic activity and increases nitrogen remobilization efficiency and grain filling. *Plant Cell Physiol.* 60 343–352. 10.1093/pcp/pcy214 30407574

[B6] Chen Z. W. Chang C. S. S. Leil T. A. Olsen R. W. (2007). C-terminal modification is required for GABARAP-mediated GABAA receptor trafficking. *J. Neurosci.* 27 6655–6663. 10.1523/jneurosci.0919-07.2007 17581952 PMC6672693

[B7] Chung T. Phillips A. R. Vierstra R. D. (2010). ATG8 lipidation and ATG8-mediated autophagy in Arabidopsis require ATG12 expressed from the differentially controlled ATG12A AND ATG12B loci. *Plant J.* 62 483–493. 10.1111/j.1365-313X.2010.04166.x 20136727

[B8] Chung T. Suttangkakul A. Vierstra R. D. (2009). The ATG autophagic conjugation system in maize: ATG transcripts and abundance of the ATG8-lipid adduct are regulated by development and nutrient availability. *Plant Physiol.* 149 220–234. 10.1104/pp.108.126714 18790996 PMC2613746

[B9] Coyle J. E. Qamar S. Rajashankar K. R. Nikolov D. B. (2002). Structure of GABARAP in two conformations: implications for GABAA receptor localization and tubulin binding. *Neuron* 33 63–74. 10.1016/S0896-6273(01)00558-X 11779480

[B10] Dagdas Y. F. Belhaj K. Maqbool A. Chaparro-Garcia A. Pandey P. Petre B. (2016). An effector of the Irish potato famine pathogen antagonizes a host autophagy cargo receptor. *eLife* 5:e10856. 10.7554/eLife.10856 26765567 PMC4775223

[B11] Deosaran E. Larsen K. B. Hua R. Sargent G. Wang Y. Kim S. (2013). NBR1 acts as an autophagy receptor for peroxisomes. *J. Cell Sci.* 126 939–952. 10.1242/jcs.114819 23239026

[B12] Dikic I. (2017). Proteasomal and autophagic degradation systems. *Annu. Rev. Biochem.* 86 193–224. 10.1146/annurev-biochem-061516-044908 28460188

[B13] Doelling J. H. Walker J. M. Friedman E. M. Thompson A. R. Vierstra R. D. (2002). The APG8/12-activating enzyme APG7 is required for proper nutrient recycling and senescence in *Arabidopsis thaliana*. *J. Biol. Chem.* 277 33105–33114. 10.1074/jbc.M204630200 12070171

[B14] Farmer L. M. Book A. J. Lee K.-H. Lin Y.-L. Fu H. Vierstra R. D. (2010). The RAD23 family provides an essential connection between the 26S proteasome and ubiquitylated proteins in *Arabidopsis*. *Plant Cell* 22 124–142. 10.1105/tpc.109.072660 20086187 PMC2828702

[B15] Farre J. C. Burkenroad A. Burnett S. F. Subramani S. (2013). Phosphorylation of mitophagy and pexophagy receptors coordinates their interaction with Atg8 and Atg11. *EMBO Rep.* 14 441–449. 10.1038/embor.2013.40 23559066 PMC3642380

[B16] Green F. O’Hare T. Blackwell A. Enns C. A. (2002). Association of human transferrin receptor with GABARAP. *FEBS Lett.* 518 101–106. 10.1016/s0014-5793(02)02655-8 11997026

[B17] Guillaumot D. Guillon S. Déplanque T. Vanhee C. Gumy C. Masquelier D. (2009). The Arabidopsis TSPO-related protein is a stress and abscisic acid-regulated, endoplasmic reticulum–Golgi-localized membrane protein. *Plant J.* 60 242–256. 10.1111/j.1365-313X.2009.03950.x 19548979

[B18] Hachez C. Veljanovski V. Reinhardt H. Guillaumot D. Vanhee C. Chaumont F. (2014). The *Arabidopsis* abiotic stress-induced TSPO-related protein reduces cell-surface expression of the aquaporin PIP2;7 through protein-protein interactions and autophagic degradation. *Plant Cell* 26 4974–4990. 10.1105/tpc.114.134080 25538184 PMC4311218

[B19] Hafrén A. Macia J. L. Love A. J. Milner J. J. Drucker M. Hofius D. (2017). Selective autophagy limits cauliflower mosaic virus infection by NBR1-mediated targeting of viral capsid protein and particles. *Proc. Natl. Acad. Sci. U.S.A.* 114 E2026–E2035. 10.1073/pnas.1610687114 28223514 PMC5347569

[B20] Hafrén A. Üstün S. Hochmuth A. Svenning S. Johansen T. Hofius D. (2018). Turnip mosaic virus counteracts selective autophagy of the viral silencing suppressor HCpro. *Plant Physiol.* 176 649–662. 10.1104/pp.17.01198 29133371 PMC5761789

[B21] Hanaoka H. Noda T. Shirano Y. Kato T. Hayashi H. Shibata D. (2002). Leaf senescence and starvation-induced chlorosis are accelerated by the disruption of an Arabidopsis autophagy gene. *Plant Physiol.* 129 1181–1193. 10.1104/pp.011024 12114572 PMC166512

[B22] Haxim Y. Ismayil A. Jia Q. Wang Y. Zheng X. Chen T. (2017). Autophagy functions as an antiviral mechanism against geminiviruses in plants. *eLife* 6:e23897. 10.7554/eLife.23897 28244873 PMC5362266

[B23] Hirata E. Ohya Y. Suzuki K. (2017). Atg4 plays an important role in efficient expansion of autophagic isolation membranes by cleaving lipidated Atg8 in *Saccharomyces cerevisiae*. *PLoS One* 12:e0181047. 10.1371/journal.pone.0181047 28704456 PMC5509253

[B24] Honig A. Avin-Wittenberg T. Ufaz S. Galili G. (2012). A new type of compartment, defined by plant-specific Atg8-interacting proteins, is induced upon exposure of *Arabidopsis* plants to carbon starvation. *Plant Cell* 24 288–303. 10.1105/tpc.111.093112 22253227 PMC3289568

[B25] Ishida H. Yoshimoto K. Izumi M. Reisen D. Yano Y. Makino A. (2008). Mobilization of rubisco and stroma-localized fluorescent proteins of chloroplasts to the vacuole by an *ATG* gene-dependent autophagic process. *Plant Physiol.* 148 142–155. 10.1104/pp.108.122770 18614709 PMC2528122

[B26] Izumi M. Ishida H. Nakamura S. Hidema J. (2017). Entire photodamaged chloroplasts are transported to the central vacuole by autophagy. *Plant Cell* 29 377–394. 10.1105/tpc.16.00637 28123106 PMC5354188

[B27] Izumi M. Wada S. Makino A. Ishida H. (2010). The autophagic degradation of chloroplasts via rubisco-containing bodies is specifically linked to leaf carbon status but not nitrogen status in Arabidopsis. *Plant Physiol.* 154 1196–1209. 10.1104/pp.110.158519 20807997 PMC2971599

[B28] Jarvis P. López-Juez E. (2013). Biogenesis and homeostasis of chloroplasts and other plastids. *Nat. Rev. Mol. Cell Biol.* 14 787–802. 10.1038/nrm3702 24263360

[B29] Ji C. H. Kwon Y. T. (2017). Crosstalk and interplay between the ubiquitin-proteasome system and autophagy. *Mol. Cells* 40 441–449. 10.14348/molcells.2017.0115 28743182 PMC5547213

[B30] Jia M. Liu X. Xue H. Wu Y. Shi L. Wang R. (2019). Noncanonical ATG8-ABS3 interaction controls senescence in plants. *Nat. Plants* 5 212–224. 10.1038/s41477-018-0348-x 30664732 PMC6368864

[B31] Johansen T. Lamark T. (2011). Selective autophagy mediated by autophagic adapter proteins. *Autophagy* 7 279–296. 10.4161/auto.7.3.14487 21189453 PMC3060413

[B32] Jung H. Lee H. N. Marshall R. S. Lomax A. W. Yoon M. J. Kim J. (2019). NBR1 mediates selective autophagy of defective proteins in Arabidopsis. *J. Exp. Bot.* 71 73–89. 10.1093/jxb/erz404 31494674 PMC6913707

[B33] Kellner R. De la Concepcion J. C. Maqbool A. Kamoun S. Dagdas Y. F. (2017). ATG8 expansion: a driver of selective autophagy diversification? *Trends Plant Sci.* 22 204–214. 10.1016/j.tplants.2016.11.015 28038982

[B34] Ketelaar T. Voss C. Dimmock S. A. Thumm M. Hussey P. J. (2004). Arabidopsis homologues of the autophagy protein Atg8 are a novel family of microtubule binding proteins. *FEBS Lett.* 567 302–306. 10.1016/j.febslet.2004.04.088 15178341

[B35] Kirisako T. Baba M. Ishihara N. Miyazawa K. Ohsumi M. Yoshimori T. (1999). Formation process of autophagosome is traced with Apg8/Aut7p in yeast. *J. Cell Biol.* 147 435–446. 10.1083/jcb.147.2.435 10525546 PMC2174223

[B36] Kirkin V. Lamark T. Sou Y.-S. Bjørkøy G. Nunn J. L. Bruun J.-A. (2009). A role for NBR1 in autophagosomal degradation of ubiquitinated substrates. *Mol. Cell* 33 505–516. 10.1016/j.molcel.2009.01.020 19250911

[B37] Klionsky D. J. Emr S. D. (2000). Autophagy as a regulated pathway of cellular degradation. *Science* 290 1717–1721. 10.1126/science.290.5497.1717 11099404 PMC2732363

[B38] Kulich I. Pecenkova T. Sekeres J. Smetana O. Fendrych M. Foissner I. (2013). Arabidopsis exocyst subcomplex containing subunit EXO70B1 is involved in autophagy-related transport to the vacuole. *Traffic* 14 1155–1165. 10.1111/tra.12101 23944713

[B39] Kumeta H. Watanabe M. Nakatogawa H. Yamaguchi M. Ogura K. Adachi W. (2010). The NMR structure of the autophagy-related protein Atg8. *J. Biomol. NMR* 47 237–241. 10.1007/s10858-010-9420-1 20428927

[B40] Lamark T. Johansen T. (2012). Aggrephagy: selective disposal of protein aggregates by macroautophagy. *Int. J. Cell Biol.* 2012:736905. 10.1155/2012/736905 22518139 PMC3320095

[B41] Lamark T. Kirkin V. Dikic I. Johansen T. (2009). NBR1 and p62 as cargo receptors for selective autophagy of ubiquitinated targets. *Cell Cycle* 8 1986–1990. 10.4161/cc.8.13.8892 19502794

[B42] Lee D. Y. Brown E. J. (2012). Ubiquilins in the crosstalk among proteolytic pathways. *Biol. Chem.* 393 441–447. 10.1515/hsz-2012-0120 22628307

[B43] Lei Y. C. Klionsky D. J. (2019). UIM-UDS: a new interface between ATG8 and its interactors. *Cell Res.* 29 507–508. 10.1038/s41422-019-0179-y 31110250 PMC6796871

[B44] Leil T. A. Chen Z. W. Chang C. S. S. Olsen R. W. (2004). GABAA receptor-associated protein traffics GABAA receptors to the plasma membrane in neurons. *J. Neurosci.* 24 11429–11438. 10.1523/jneurosci.3355-04.2004 15601949 PMC6730371

[B45] Li F. Q. Chung T. Vierstra R. D. (2014). AUTOPHAGY-RELATED11 plays a critical role in general autophagy- and senescence-induced mitophagy in Arabidopsis. *Plant Cell* 26 788–807. 10.1105/tpc.113.120014 24563201 PMC3967041

[B46] Li F. Q. Vierstra R. D. (2012). Autophagy: a multifaceted intracellular system for bulk and selective recycling. *Trends Plant Sci.* 17 526–537. 10.1016/j.tplants.2012.05.006 22694835

[B47] Li J. Yin J. Rong C. Li K. E. Wu J. X. Huang L. Q. (2016). Orosomucoid proteins interact with the small subunit of serine palmitoyltransferase and contribute to sphingolipid homeostasis and stress responses in Arabidopsis. *Plant Cell* 28 3038–3051. 10.1105/tpc.16.00574 27923879 PMC5240739

[B48] Lin L. Yang P. Huang X. Zhang H. Lu Q. Zhang H. (2013). The scaffold protein EPG-7 links cargo–receptor complexes with the autophagic assembly machinery. *J. Cell Biol.* 201 113–129. 10.1083/jcb.201209098 23530068 PMC3613692

[B49] Liu Y. M. Bassham D. C. (2012). Autophagy: pathways for self-eating in plant cells. *Annu. Rev. Plant Biol.* 63 215–237. 10.1146/annurev-arplant-042811-105441 22242963

[B50] Lu K. Psakhye I. Jentsch S. (2014). Autophagic clearance of PolyQ proteins mediated by Ubiquitin-Atg8 adaptors of the conserved CUET protein family. *Cell* 158 549–563. 10.1016/j.cell.2014.05.048 25042851

[B51] Maqbool A. Hughes R. K. Dagdas Y. F. Tregidgo N. Zess E. Belhaj K. (2016). Structural basis of host autophagy-related protein 8 (ATG8) binding by the Irish potato famine pathogen effector protein PexRD54. *J. Biol. Chem.* 291 20270–20282. 10.1074/jbc.M116.744995 27458016 PMC5025708

[B52] Marshall R. S. Hua Z. Mali S. McLoughlin F. Vierstra R. D. (2019). ATG8-Binding UIM proteins define a new class of autophagy adaptors and receptors. *Cell* 177 766–781. 10.1016/j.cell.2019.02.009 30955882 PMC6810650

[B53] Marshall R. S. Li F. Gemperline D. C. Book A. J. Vierstra R. D. (2015). Autophagic degradation of the 26S proteasome is mediated by the dual ATG8/Ubiquitin receptor RPN10 in Arabidopsis. *Mol. Cell* 58 1053–1066. 10.1016/j.molcel.2015.04.023 26004230 PMC4903074

[B54] Marshall R. S. McLoughlin F. Vierstra R. D. (2016). Autophagic turnover of inactive 26S proteasomes in yeast is directed by the ubiquitin receptor Cue5 and the Hsp42 chaperone. *Cell Rep.* 16 1717–1732. 10.1016/j.celrep.2016.07.015 27477278

[B55] Marshall R. S. Vierstra R. D. (2018). Autophagy: the master of bulk and selective recycling. *Annu. Rev. Plant Biol.* 69 173–208. 10.1146/annurev-arplant-042817-040606 29539270

[B56] Masclaux-Daubresse C. Chen Q. Havé M. (2017). Regulation of nutrient recycling via autophagy. *Curr. Opin. Plant Biol.* 39 8–17. 10.1016/j.pbi.2017.05.001 28528166

[B57] Michaeli S. Honig A. Levanony H. Peled-Zehavi H. Galili G. (2014). Arabidopsis ATG8-INTERACTING PROTEIN1 is involved in autophagy-dependent vesicular trafficking of plastid proteins to the vacuole. *Plant Cell* 26 4084–4101. 10.1105/tpc.114.129999 25281689 PMC4247578

[B58] Minina E. A. Moschou P. N. Vetukuri R. R. Sanchez-Vera V. Cardoso C. Liu Q. (2018). Transcriptional stimulation of rate-limiting components of the autophagic pathway improves plant fitness. *J. Exp. Bot.* 69 1415–1432. 10.1093/jxb/ery010 29365132 PMC6019011

[B59] Mizushima N. (2010). The role of the Atg1/ULK1 complex in autophagy regulation. *Curr. Opin. Cell Biol.* 22 132–139. 10.1016/j.ceb.2009.12.004 20056399

[B60] Muller J. M. M. Shorter J. Newman R. Deinhardt K. Sagiv Y. Elazar Z. (2002). Sequential SNARE disassembly and GATE-16–GOS-28 complex assembly mediated by distinct NSF activities drives Golgi membrane fusion. *J. Cell Biol.* 157 1161–1173. 10.1083/jcb.200202082 12070132 PMC2173554

[B61] Nair U. Yen W. L. Mari M. Cao Y. Xie Z. Baba M. (2012). A role for Atg8–PE deconjugation in autophagosome biogenesis. *Autophagy* 8 780–793. 10.4161/auto.19385 22622160 PMC3378420

[B62] Nakamura S. Hidema J. Sakamoto W. Ishida H. Izumi M. (2018). Selective elimination of membrane-damaged chloroplasts via microautophagy. *Plant Physiol.* 177 1007–1026. 10.1104/pp.18.00444 29748433 PMC6052986

[B63] Nakamura T. Hayashi T. Nasu-Nishimura Y. Sakaue F. Morishita Y. Okabe T. (2008). PX-RICS mediates ER-to-Golgi transport of the N-cadherin/β-catenin complex. *Genes Dev.* 22 1244–1256. 10.1101/gad.1632308 18451111 PMC2335319

[B64] Nakatogawa H. Ichimura Y. Ohsumi Y. (2007). Atg8, a ubiquitin-like protein required for autophagosome formation, mediates membrane tethering and hemifusion. *Cell* 130 165–178. 10.1016/j.cell.2007.05.021 17632063

[B65] Noda N. N. Ohsumi Y. Inagaki F. (2010). Atg8-family interacting motif crucial for selective autophagy. *FEBS Lett.* 584 1379–1385. 10.1016/j.febslet.2010.01.018 20083108

[B66] Nolan T. M. Brennan B. Yang M. R. Chen J. N. Zhang M. C. Li Z. H. (2017). Selective autophagy of BES1 mediated by DSK2 balances plant growth and survival. *Dev. Cell* 41 33–46. 10.1016/j.devcel.2017.03.013 28399398 PMC5720862

[B67] Ohsumi Y. (2001). Molecular dissection of autophagy: two ubiquitin-like systems. *Nat. Rev. Mol. Cell Biol.* 2 211–216. 10.1038/35056522 11265251

[B68] Pankiv S. Alemu E. A. Brech A. Bruun J. A. Lamark T. Øvervatn A. (2010). FYCO1 is a Rab7 effector that binds to LC3 and PI3P to mediate microtubule plus end–directed vesicle transport. *J. Cell Biol.* 188 253–269. 10.1083/jcb.200907015 20100911 PMC2812517

[B69] Pankiv S. Clausen T. H. Lamark T. Brech A. Bruun J. A. Outzen H. (2007). p62/SQSTM1 binds directly to Atg8/LC3 to facilitate degradation of ubiquitinated protein aggregates by autophagy. *J. Biol. Chem.* 282 24131–24145. 10.1074/jbc.M702824200 17580304

[B70] Perez-Perez M. E. Crespo J. L. (2010). Autophagy in the model alga Chlamydomonas reinhardtii. *Autophagy* 6 562–563. 10.4161/auto.6.4.11822 20404489

[B71] Phillips A. R. Suttangkakul A. Vierstra R. D. (2008). The ATG12-conjugating enzyme ATG10 is essential for autophagic vesicle formation in *Arabidopsis thaliana*. *Genetics* 178 1339–1353. 10.1534/genetics.107.086199 18245858 PMC2278079

[B72] Ren W. Liu N. Sang C. Shi D. Zhou M. Chen C. (2018). The autophagy gene BcATG8 regulates the vegetative differentiation and pathogenicity of *Botrytis cinerea*. *Appl. Environ. Microbiol.* 84:e02455-17. 10.1128/aem.02455-17 29572212 PMC5960959

[B73] Sagiv Y. Legesse-Miller A. Porat A. Elazar Z. (2000). GATE-16, a membrane transport modulator, interacts with NSF and the Golgi v-SNARE GOS-28. *EMBO J.* 19 1494–1504. 10.1093/emboj/19.7.1494 10747018 PMC310219

[B74] Sargent G. van Zutphen T. Shatseva T. Zhang L. Di Giovanni V. Bandsma R. (2016). PEX2 is the E3 ubiquitin ligase required for pexophagy during starvation. *J. Cell Biol.* 214 677–690. 10.1083/jcb.201511034 27597759 PMC5021090

[B75] Seo E. Woo J. Park E. Bertolani S. J. Siegel J. B. Choi D. (2016). Comparative analyses of ubiquitin-like ATG8 and cysteine protease ATG4 autophagy genes in the plant lineage and cross-kingdom processing of ATG8 by ATG4. *Autophagy* 12 2054–2068. 10.1080/15548627.2016.1217373 27540766 PMC5103345

[B76] Shpilka T. Weidberg H. Pietrokovski S. Elazar Z. (2011). Atg8: an autophagy-related ubiquitin-like protein family. *Genome Biol.* 12:226. 10.1186/gb-2011-12-7-226 21867568 PMC3218822

[B77] Signorelli S. Tarkowski Ł. P. Van den Ende W. Bassham D. C. (2019). Linking autophagy to abiotic and biotic stress responses. *Trends Plant Sci.* 24 413–430. 10.1016/j.tplants.2019.02.001 30824355 PMC6475611

[B78] Sjogaard I. M. Z. Bressendorff S. Prestel A. Kausika S. Oksbjerg E. Kragelund B. B. (2019). The transmembrane autophagy cargo receptors ATI1 and ATI2 interact with ATG8 through intrinsically disordered regions with distinct biophysical properties. *Biochem. J.* 476 449–465. 10.1042/bcj20180748 30642888

[B79] Slavikova S. Shy G. Yao Y. L. Giozman R. Levanony H. Pietrokovski S. (2005). The autophagy-associated Atg8 gene family operates both under favourable growth conditions and under starvation stresses in Arabidopsis plants. *J. Exp. Bot.* 56 2839–2849. 10.1093/jxb/eri276 16157655

[B80] Su T. Li X. Yang M. Shao Q. Zhao Y. Ma C. (2020). Autophagy: an intracellular degradation pathway regulating plant survival and stress response. *Front. Plant Sci.* 11:164. 10.3389/fpls.2020.00164 32184795 PMC7058704

[B81] Sugawara K. Suzuki N. N. Fujioka Y. Mizushima N. Ohsumi Y. Inagaki F. (2004). The crystal structure of microtubule-associated protein light chain 3, a mammalian homologue of *Saccharomyces cerevisiae* Atg8. *Genes Cells* 9 611–618. 10.1111/j.1356-9597.2004.00750.x 15265004

[B82] Suttangkakul A. Li F. Q. Chung T. Vierstra R. D. (2011). The ATG1/ATG13 protein kinase complex is both a regulator and a target of autophagic recycling in Arabidopsis. *Plant Cell* 23 3761–3779. 10.1105/tpc.111.090993 21984698 PMC3229148

[B83] Svenning S. Lamark T. Krause K. Johansen T. (2011). Plant NBR1 is a selective autophagy substrate and a functional hybrid of the mammalian autophagic adapters NBR1 and p62/SQSTM1. *Autophagy* 7 993–1010. 10.4161/auto.7.9.16389 21606687 PMC3210314

[B84] Teh O. K. Lee C. W. Ditengou F. A. Klecker T. Furlan G. Zietz M. (2018). Phosphorylation of the exocyst subunit Exo70B2 contributes to the regulation of its function. *bioRxiv* [Preprint]. 10.1101/266171

[B85] Thompson A. R. Doelling J. H. Suttangkakul A. Vierstra R. D. (2005). Autophagic nutrient recycling in Arabidopsis directed by the ATG8 and ATG12 conjugation pathways. *Plant Physiol.* 138 2097–2110. 10.1104/pp.105.060673 16040659 PMC1183398

[B86] Thompson A. R. Vierstra R. D. (2005). Autophagic recycling: lessons from yeast help define the process in plants. *Curr. Opin. Plant Biol.* 8 165–173. 10.1016/j.pbi.2005.01.013 15752997

[B87] Tzfadia O. Galili G. (2013). The Arabidopsis exocyst subcomplex subunits involved in a golgi-independent transport into the vacuole possess consensus autophagy-associated atg8 interacting motifs. *Plant Signal. Behav.* 8:e26732. 10.4161/psb.26732 24494242 PMC4091113

[B88] Üstün S. Hafrén A. Liu Q. Marshall R. S. Minina E. A. Bozhkov P. V. (2018). Bacteria exploit autophagy for proteasome degradation and enhanced virulence in plants. *Plant Cell* 30 668–685. 10.1105/tpc.17.00815 29500318 PMC5894834

[B89] Vanhee C. Zapotoczny G. Masquelier D. Ghislain M. Batoko H. (2011). The Arabidopsis multistress regulator TSPO is a heme binding membrane protein and a potential scavenger of porphyrins via an autophagy-dependent degradation mechanism. *Plant Cell* 23 785–805. 10.1105/tpc.110.081570 21317376 PMC3077796

[B90] Wang Z. Li X. Liu N. Peng Q. Wang Y. Fan B. (2019). A family of NAI2-interacting proteins in the biogenesis of the ER body and related structures. *Plant Physiol.* 180 212–227. 10.1104/pp.18.01500 30770459 PMC6501091

[B91] Woo J. Park E. Dinesh-Kumar S. P. (2014). Differential processing of Arabidopsis ubiquitin-like Atg8 autophagy proteins by Atg4 cysteine proteases. *Proc. Natl. Acad. Sci. U.S.A.* 111 863–868. 10.1073/pnas.1318207111 24379391 PMC3896200

[B92] Wywial E. Singh S. M. (2010). Identification and structural characterization of FYVE domain-containing proteins of *Arabidopsis thaliana*. *BMC Plant Biol.* 10:157. 10.1186/1471-2229-10-157 20678208 PMC3017826

[B93] Xia K. Liu T. Ouyang J. Wang R. Fan T. Zhang M. (2011). Genome-wide identification, classification, and expression analysis of autophagy-associated gene homologues in rice (*Oryza sativa* L.). *DNA Res.* 18 363–377. 10.1093/dnares/dsr024 21795261 PMC3190957

[B94] Xia T. Xiao D. Liu D. Chai W. Gong Q. Wang N. N. (2012). Heterologous expression of ATG8c from soybean confers tolerance to nitrogen deficiency and increases yield in Arabidopsis. *PLoS One* 7:e37217. 10.1371/journal.pone.0037217 22629371 PMC3358335

[B95] Xie Q. Tzfadia O. Levy M. Weithorn E. Peled-Zehavi H. Van Parys T. (2016). hfAIM: a reliable bioinformatics approach for in silico genome-wide identification of autophagy-associated Atg8-interacting motifs in various organisms. *Autophagy* 12 876–887. 10.1080/15548627 27071037 PMC4854547

[B96] Xie Z. Nair U. Klionsky D. J. (2008). Atg8 controls phagophore expansion during autophagosome formation. *Mol. Biol. Cell* 19 3290–3298. 10.1091/mbc.E07-12-1292 18508918 PMC2488302

[B97] Yang F. Kimberlin A. N. Elowsky C. G. Liu Y. Gonzalez-Solis A. Cahoon E. B. (2019). A plant immune receptor degraded by selective autophagy. *Mol. Plant* 12 113–123. 10.1016/j.molp.2018.11.011 30508598

[B98] Yoshimoto K. Hanaoka H. Sato S. Kato T. Tabata S. Noda T. (2004). Processing of ATG8s, ubiquitin-like proteins, and their deconjugation by ATG4s are essential for plant autophagy. *Plant Cell* 16 2967–2983. 10.1105/tpc.104.025395 15494556 PMC527192

[B99] Yu J. Zhen X. Li X. Li N. Xu F. (2019). Increased autophagy of rice can increase yield and Nitrogen Use Efficiency (NUE). *Front. Plant Sci.* 10:584. 10.3389/fpls.2019.00584 31134120 PMC6514234

[B100] Yu Z. Q. Ni T. Hong B. Wang H.-Y. Jiang F. J. Zou S. (2012). Dual roles of Atg8-PE deconjugation by Atg4 in autophagy. *Autophagy* 8 883–892. 10.4161/auto.19652 22652539 PMC3427254

[B101] Zess E. K. Jensen C. Cruz-Mireles N. De la Concepcion J. C. Sklenar J. Stephani M. (2019). N-terminal β-strand underpins biochemical specialization of an ATG8 isoform. *PLoS Biol.* 17:e3000373. 10.1371/journal.pbio.3000373 31329577 PMC6675122

[B102] Zhan N. Wang C. Chen L. C. Yang H. J. Feng J. Gong X. Q. (2018). S-Nitrosylation Targets GSNO reductase for selective autophagy during hypoxia responses in plants. *Mol. Cell* 71 142–154. 10.1016/j.molcel.2018.05.024 30008318

[B103] Zhang J. Tripathi D. N. Jing J. Alexander A. Kim J. Powell R. T. (2015). ATM functions at the peroxisome to induce pexophagy in response to ROS. *Nat. Cell Biol.* 17 1259–1269. 10.1038/ncb3230 26344566 PMC4589490

[B104] Zhou J. Wang J. Cheng Y. Chi Y. J. Fan B. Yu J. Q. (2013). NBR1-mediated selective autophagy targets insoluble ubiquitinated protein aggregates in plant stress responses. *PLoS Genet.* 9:e1003196. 10.1371/journal.pgen.1003196 23341779 PMC3547818

[B105] Zhou J. Wang J. Yu J. Q. Chen Z. (2014). Role and regulation of autophagy in heat stress responses of tomato plants. *Front. Plant Sci.* 5:174. 10.3389/fpls.2014.00174 24817875 PMC4012191

[B106] Zhou J. Wang Z. Wang X. Li X. Zhang Z. Fan B. (2018). Dicot-specific ATG8-interacting ATI3 proteins interact with conserved UBAC2 proteins and play critical roles in plant stress responses. *Autophagy* 14 487–504. 10.1080/15548627.2017.1422856 29313416 PMC5915045

[B107] Zhuang X. Jiang L. (2019). Chloroplast degradation: multiple routes into the vacuole. *Front. Plant Sci.* 10:359. 10.3389/fpls.2019.00359 30972092 PMC6443708

[B108] Zhuang X. Chung K. P. Cui Y. Lin W. Gao J. Kang B. H. (2017). ATG9 regulates autophagosome progression from the endoplasmic reticulum in *Arabidopsis*. *Proc. Natl. Acad. Sci. U. S. A.* 114, E426–E435. 10.1073/pnas.1616299114 28053229 PMC5255614

[B109] Zhuang X. Chung K. P. Luo M. Jiang L. (2018). Autophagosome biogenesis and the endoplasmic reticulum: a plant perspective. *Trends Plant Sci.* 23 677–692. 10.1016/j.tplants.2018.05.002 29929776

[B110] Zhuang X. Wang H. Lam S. K. Gao C. Wang X. Cai Y. (2013). A BAR-domain protein SH3P2, which binds to phosphatidylinositol 3-phosphate and ATG8, regulates autophagosome formation in Arabidopsis. *Plant Cell* 25 4596–4615. 10.1105/tpc.113.118307 24249832 PMC3875738

[B111] Zientara-Rytter K. Lukomska J. Moniuszko G. Gwozdecki R. Surowiecki P. Lewandowska M. (2011). Identification and functional analysis of Joka2, a tobacco member of the family of selective autophagy cargo receptors. *Autophagy* 7 1145–1158. 10.4161/auto.7.10.16617 21670587 PMC3242614

[B112] Zientara-Rytter K. Ozeki K. Nazarko T. Y. Subramani S. (2018). Pex3 and Atg37 compete to regulate the interaction between the pexophagy receptor, Atg30, and the Hrr25 kinase. *Autophagy* 14 368–384. 10.1080/15548627.2017.1413521 29260977 PMC5915033

