# Supplementary material for: Multiple Functions of ATG8 Family Proteins in Plant Autophagy
Source: Front Cell Dev Biol. 2020 Jun 10;8:466. doi: 10.3389/fcell.2020.00466 (PMC7301642; doi:10.3389/fcell.2020.00466)
Supplement: Supplementary file 1 [file Table_1.DOCX]

Table 1. ATG8 Gene Family in Plants

| **Species** | **Common name** | **Number** | **Gene name** | **Locus** | **Reference** |
| --- | --- | --- | --- | --- | --- |
| *Chlamydomonas reinhardtii* | Green algae | 1 | CrATG8 | Cre16.g689650 | Perez-Perez and Crespo, 2010 |
| *Zea mays* | Maize | 5 | ZmATG8a  ZmATG8b  ZmATG8c  ZmATG8d  ZmATG8e | GRMZM2G336871  GRMZM2G419694  GRMZM2G076826  GRMZM2G134613  GRMZM2G014975 | Chung et al., 2009 |
| *Oryza sativa* | Rice | 7 | OsATG8a  OsATG8b  OsATG8c  OsATG8d  OsATG8e  OsATG8f  OsATG8i | Os07g0512200  Os04g0624000  Os08g0191600  Os02g0529150  Os11g0100100  Os12g0100050  NA | Chung et al., 2009; Xia et al., 2011 |
| *Solanum tuberosum* | Potato | 7 | StATG8-1.1  StATG8-1.2  StATG8-2.1  StATG8-2.2  StATG8-3.1  StATG8-3.2  StATG8-4 | PGSC0003DMP400008510  PGSC0003DMP400022074  PGSC0003DMP400038670  PGSC0003DMP400025414  PGSC0003DMP400028821  PGSC0003DMP400009039  PGSC0003DMP400009229 | Maqbool et al., 2016; Kellner et al., 2017; Zess et al., 2019 |
| *Arabidopsis thaliana* | Arabidopsis | 9 | AtATG8a  AtATG8b  AtATG8c  AtATG8d  AtATG8e  AtATG8f  AtATG8g  AtATG8h  AtATG8i | At4G21980  At4G04620  At1G62040  At2G05630  At2G45170  At4G16520  At3G60640  At3G06420  At3G15580 | Doelling et al., 2002; Hanaoka et al., 2002 |
| *Glycine max* | Soybean | 11 | GmATG8a  GmATG8b  GmATG8c  GmATG8d  GmATG8e  GmATG8f  GmATG8g  GmATG8h  GmATG8i  GmATG8j  GmATG8k | Gm17g01650  Gm07g39090  Gm09g00630  Gm12g10510  Gm06g46270  Gm05g04540  Gm17g14970  Gm10g01220  Gm02g01180  Gm11g03460  Gm01g41910 | Xia et al., 2012 |
